# The Effects of an 8-Month Multicomponent Training Program in Body Composition, Functional Fitness, and Sleep Quality in Aged People: A Randomized Controlled Trial

**DOI:** 10.3390/jcm13216603

**Published:** 2024-11-03

**Authors:** Pedro Forte, Samuel G. Encarnação, Luís Branquinho, Tiago M. Barbosa, António M. Monteiro, Daniel Pecos-Martín

**Affiliations:** 1 Physiotherapy and Pain Group, Department of Physical Therapy, University of Alcala, 28801 Madrid, Spain; daniel.pecos@uah.es; 2 Department of Sports, Higher Institute of Educational Sciences of the Douro, 4560-708 Penafiel, Portugal; 3 Research Centre for Active Living and Wellbeing (LiveWell), Instituto Politécnico de Bragança, 5300-253 Bragança, Portugal; 4 Department of Sports Sciences, Instituto Politécnico de Bragança, 5300-253 Bragança, Portugal; 5 Department of Physical Activity and Sport Sciences, Universidad Autónoma de Madrid (UAM), 28049 Madrid, Spain; 6 Biosciences Higher School of Elvas, Polytechnic Institute of Portalegre, 7350-092 Portalegre, Portugal; 7 Life Quality Research Centre (LORQ-CIEQV), 2001-964 Santarém, Portugal; 8 Research Center in Sport Sciences, Health Sciences and Human Development (CIDESD), 6201-001 Covilhã, Portugal

**Keywords:** ageing, fitness, sleep, quality

## Abstract

**Background/Objectives**: This study examined the effects of an intervention on anthropometrics, body composition, physical fitness, and sleep quality in aged individuals, comparing a control group (N = 11) and an experimental group (N = 13) across two measurement points. **Methods**: A multicomponent training program of 8 months was adopted as the intervention group. A bioimpedance balance, functional fitness test, and Pittsburgh Sleep Quality Index measured body composition, functional fitness, and sleep quality. **Results**: Both groups showed minimal changes in body mass and hand grip strength. However, the experimental group experienced significant improvements in physical fitness, including a 26% increase in arm curl repetitions, an 18% reduction in 5 times sit-to-stand (5TSTS) completion time, and a 29% rise in 2-min step test (2MST) steps, indicating enhanced muscle endurance and cardiovascular fitness. Flexibility decreased significantly in the experimental group, while body fat percentage was reduced by 10%. Sleep quality improved by 47% in the experimental group but declined by 14% in the control group. Correlational analysis revealed that better sleep quality was linked to improved fitness performance and reduced body fat in the experimental group, with post-intervention results further confirming the connection between sleep and fat reduction. In the control group, improved sleep quality was associated with higher metabolic rates after 8 months. **Conclusions**: These findings suggest that the intervention positively impacted physical fitness and sleep quality, with potential benefits for overall health.

## 1. Introduction

The elderly commonly experience deterioration in the visual and proprioceptive systems, which can impact balance and postural control [1,2]. In older adults, proprioceptive information plays an important role in maintaining balance. Other studies [3,4] have underscored the role of balance training in improving proprioception and dynamic balance. The balance training type can enhance ankle stability (i.e., lower limbs function), neuromuscular function (i.e., related to strength), and postural control system efficiency [5,6]. This highlights the significance of incorporating balance exercises targeting proprioception in older adults to enhance dynamic balance and reduce the risk of falls [7]. Finally, [8] identified a link between sleep quality and dynamic balance, indicating that poor sleep quality may be associated with dynamic imbalance in older adults. However, the authors were unable to evaluate the effects of training exercise programs on physical fitness, sleep quality, and balance.

The relationship between sleep quality and physical exercise in older people has been widely documented in the literature. Studies have shown that aerobic exercise can improve self-reported sleep and quality of life in older adults with insomnia [9]. Additionally, systematic reviews have indicated that physical activity programs positively impact various aspects of sleep in generally healthy older adults [10]. A study [11] showed that in community-dwelling older adults, exercise can impact sleep through mechanisms such as light exposure, temperature regulation, and mood. A recent systematic review and meta-analysis have further supported the positive effects of physical exercise programs on improving sleep quality in older adults [12]. However, a longitudinal study demonstrated that sleep quality plays a crucial role in older adults’ level of physical activity, with better sleep quality promoting more physical activity [13]. Older individuals often struggle with changing positions during sleep due to musculoskeletal pain, decreased mobility, and motor impairments [14,15], leading to discomfort and sleep disturbances [16]. Also, chronic pain conditions and limited ability to change positions worsen sleep difficulties [17], compounded by physical disabilities and cognitive impairments [15]. Poor sleep quality is independently linked to physical disability and functional limitations in older adults [18,19], stressing the need to address sleep disturbances to enhance overall health and well-being [18,20]. Upon that, physical exercise training programs may improve body functionality and possibly sleep quality. Between the different exercise training programs, the American College of Sports Medicine (ACSM) previously highlighted the importance of multicomponent training (MCT) for older adults, citing its benefits for strength, aerobic fitness, and balance [21]. This type of program (MCT) including exercises for aerobics, resistance, balance, and flexibility composes the multicomponent training [22,23]. It could improve metabolic outcomes, functional and cognitive performance, cardiorespiratory fitness and autonomy. The fundamental part of each training session aims to train aerobic, resistance, and balance skills [22,23]. Subsequent research has supported these claims. A review of 27 studies showed that MCT improves physical fitness and overall health in older populations [24]. Additionally, a meta-analysis comparing aerobic training, resistance training, and MCT found MCT to be the most effective for cognitive improvement [25]. Recent research also supports MCT’s positive effects on attention and executive function in older adults [26]. MCT programs include exercises targeting physical and cognitive health [5,27,28], aiming to enhance muscle mass, power output, functional outcomes, cognitive function, and brain health [6,29]. Overall, MCT appears effective in improving physical function, muscle mass, cognitive function, and mental health in older adults, even those with conditions like mild cognitive impairment, dementia, Alzheimer’s disease, and sarcopenia [28,30].

The relationship between exercise, physical fitness, body composition, and sleep quality in older adults may be complex and interconnected [31,32]. In light of the above, it seems important to continue investigating to understand the complexity of the phenomenon to broadly understand the effects of exercise on sleep quality and its associations with physical fitness and body composition, which can play a fundamental role in the development of interventions that promote healthy aging and improve the well-being of older individuals. Given this, this research aimed to evaluate the effects of 8 months of multicomponent training programs on critical variables such as physical fitness, body composition, and sleep quality. Additionally, we aim to seek the associations between physical fitness, body composition, and sleep quality. It was hypothesized that an 8-month multicomponent training program would significantly positively affect physical fitness, body composition, and sleep quality.

## 2. Materials and Methods

### 2.1. Design and Sample

**Figure 1:**
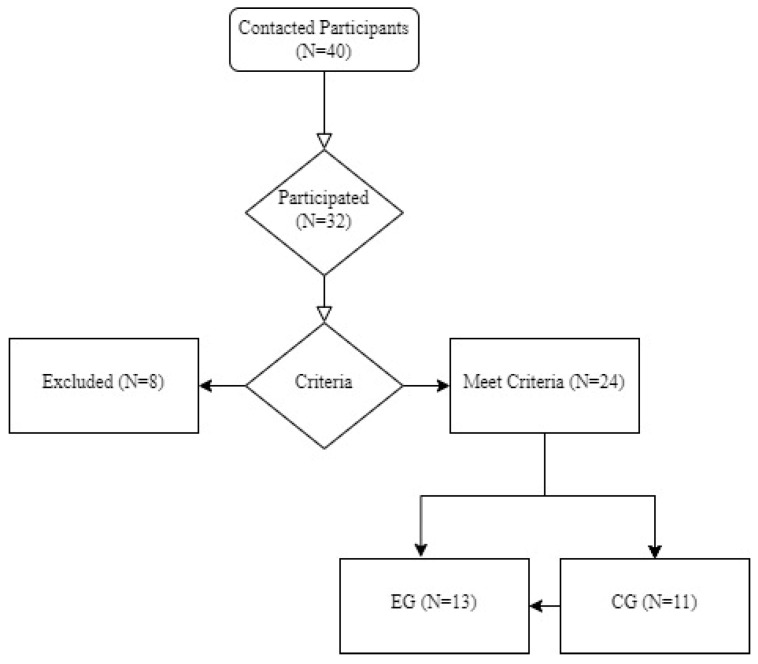
Participants flowchart of sampling.

This was a randomized controlled trial with pre- and post-intervention measurements (The trial was registered with the ID NCT06646380 on the ClinicalTrials.gov: https://clinicaltrials.gov/study/NCT06646380). Forty participants were contacted to participate in this study and randomly divided into two groups of 20 participants (Control and Experimental Groups). The convenience sample’s mean age was 69 years old. The sample was recruited in the Bragança Municipality in Portugal. All participants were aged community people. All procedures were carried out in accordance with the recommendations of the Declaration of Helsinki for human studies. The research project received approval by the Ethical Committee of the Instituto Politécnico de Bragança (number: 2576). The participants were instructed to maintain normal daily activities to prevent physical inactivity. The participants were asked to complete a sample characterization questionnaire during the first visit. The criteria for inclusion in the study were: (i) being aged 65 years or older, (ii) maintaining functional independence in daily tasks, (iii) having no severe chronic diseases or medications to sleep that could affect the results, (iv) do not have significant cardiovascular, muscular, metabolic, or joint complications. Among the 40 contacted participants, only 32 subjects completed the first evaluation (15 were from the experimental group (EG) and 17 were from the control group (CG)). Eight participants (25% of the total sample) dropped out due to undeclared health issues and losing interest in the study. Between them, 2 participants from the experimental group did not attend the minimum of 75% of the exercise sessions. Thus, the data from the remaining participants (13 in EG: 11 women and 2 men) were not involved in any physical exercise or similar intervention during all follow-ups. The CG (11: 9 women and 2 men) were instructed to maintain daily routines. However, the typical profile of this group was that the participants were regularly physically active people. Most participate in municipal activities like nature walking, physical activity sessions including dance (casually), board and card games, and traditional games (Bocce, Adapted Bowling, and darts). After 32 weeks and in the second moment, only 24 participants finalized the study (13 from EG and 11 from CG). Figure 1 depicts the sampling flowchart of the sampling process.

### 2.2. Intervention Program

**Figure 2:**
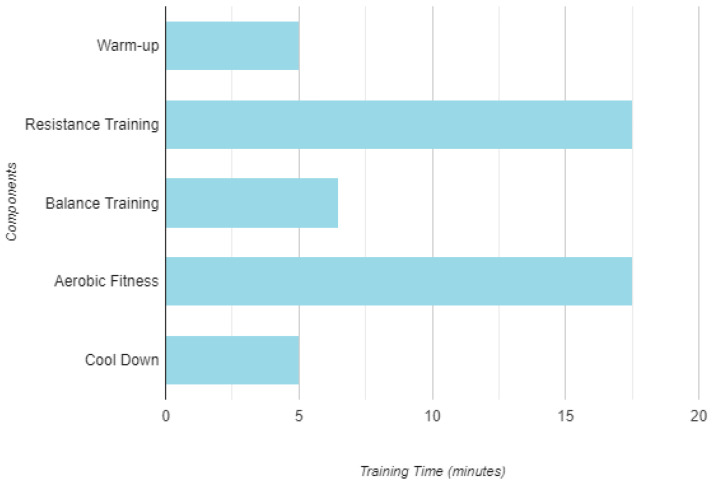
Multicomponent training component volume.

The exercise program integrated aerobic, resistance, flexibility, and balance activities [22,23]. Each session lasted between 50 and 60 min and consisted of five key components (Figure 2): (i) a 5–8 min warm-up with slow walking and stretching; (ii) 15–20 min of aerobic activities, including walking, jogging, aerobics, and dancing, with at least 8–10 min; and (iii) 1–3 sets of resistance exercises using elastic bands and free weights in a circuit format, targeting major muscle groups, such as knee flexors/extensors, shoulder abductors/adductors, elbow flexors/extensors, pectorals, and abdominals, with 40–60 s of rest between sets. To ensure proper familiarization and technique, the training intensity started lower at the beginning of each month, beginning with 8 repetitions in 1 set and gradually increasing to 12–15 repetitions and 3 sets; (iv) 5–8 min of static and dynamic balance training using sticks, balls, and balloons; and (v) a 5-min cool-down period at the end of each session, including breathing exercises and stretching.

The repetitions and time increased by 30% after each 2-month intervention. The training intensity was controlled using Borg’s 10-point categoric ratio scale (CR-10) [33]. The coaches aimed to work in an intensity range from 3 (moderate) to 5 (intense). Table 1 presents the training session plan.

### 2.3. Anthropometrics and Body Composition

Anthropometric measurements included height and weight. Body composition was assessed using a digital bioimpedance scale (Tanita BC-50, IL, USA), which recorded variables such as lean mass, body fat percentage, bone mineral density, visceral fat, total body mass, muscle mass, fat mass, and bone density. Evaluations were conducted in the morning before breakfast, with participants wearing only light clothing and no shoes or socks. Height was measured while the participants stood with their head aligned in the Frankfurt plane. Waist and hip circumferences were also measured. Metabolic variables, including systolic and diastolic blood pressure and resting heart rate, were recorded using an OMRON (M2 HEM-7143-E) and brachial cuff (Easy 22–32) (Amsterdam, The Netherlands). The metabolic rate was assessed via bioimpedance using the Tanita scale.

### 2.4. Physical Fitness

Handgrip strength was assessed using a digital palmar dynamometer (CAMRY^®^, Lisbon, Portugal), with the maximum kilograms-force (kgf) achieved using a palm grip as the measurement. The participants stood with their arms away from their body and, upon the researcher’s signal, exerted maximum palm grip force on the dynamometer for 4 s [34]. Each participant was given three attempts, and the highest recorded result was noted by the evaluator.

The Functional Fitness Test was used to assess the main physical parameters associated with functional mobility. The Functional Fitness Test evaluated key aspects of physical mobility [35]. It included the 2-Minute Step Test, where the evaluator set a knee height marker using a measuring tape to measure from the kneecap to the iliac crest, and participants aimed to take as many steps as possible in 2 minutes, with performance checks at 60 and 90 s. The Seat-to-Stand Test required participants to repeatedly sit and stand from a 43-cm highchair for 30 s, with the number of repetitions recorded. In the Arm Curl Test, participants seated on a 43-cm chair used a 2-kg dumbbell to perform elbow curls for 30 s. The Time-Up-and-Go Test involved starting from a seated position on a 43-cm chair, walking quickly around a cone placed 2.44 m away, and returning to the chair, with the time recorded after 2 attempts. The Sit and Reach Test, conducted while seated on a 43-cm chair with one leg extended and toes reached, and the Back Scratch Test, where participants attempted to touch one hand with the other behind their back, were timed in seconds.

The lower limb muscle power was assessed using the five-time sit-to-stand test with a chair 0.49 m high. The evaluator started the stopwatch when the participant stood up and stopped it after completing five repetitions, marking the time as soon as they sat back down for the fifth time. The evaluator encouraged the participant to maintain maximum speed and proper technique throughout the test. Each participant performed 2 attempts, with a 60-s rest between them, and the shortest time was recorded [36].

### 2.5. Sleep Quality

Sleep quality was assessed using the Pittsburgh Sleep Quality Index (PSQI), a 19-item questionnaire developed by Buysse et al. [37] and validated for the Portuguese population [38]. The PSQI items are divided into the following components: (1) subjective sleep quality, (2) sleep latency, (3) sleep duration, (4) habitual sleep efficiency, (5) sleep disturbances, (6) use of sleeping medication, and (7) daytime dysfunction. Each item is scored from 0 to 3, with the total score ranging from 0 to 21; higher scores indicate more severe sleep disturbances. A global score of 5 or higher in 2 components signifies significant sleep difficulties, while a score in 3 or more components indicates moderate sleep issues. A total score below 5 indicates good sleep quality, whereas a score above 5 indicates poor sleep quality.

### 2.6. Statistical Analysis

The analysis of the sample statistical power was classified as reduced of the prior recruited sample in this study (<0.80). The standard statistical methods were used to calculate the means and standard deviation. The Kolmogorov–Smirnov test allowed us to assess the normality of the distribution, and Levene’s test assessed the homogeneity (N < 30).

The t-test permitted the comparison of variables by sex and sleep quality, and Pearson’s correlation permitted the test of the association between the variables for the control and the experimental group, respectively. The test was made at a significance level of 5%. Effect sizes were calculated based on Cohen’s d and classified as 0.2—trivial; 0.6—small; 1.2—large; and>2.0—very large [39]. Statistical analyses were performed with 95% CI; *p* < 0.05. All procedures were performed with SPSS version 24.0 (SPSS, Inc., Chicago, IL, USA). Additionally, we applied statistical procedures based on Bayesian assumptions. For this purpose, a two-way mixed effect Bayesian ANOVA was applied to capture effects within, between, and interactions between within subjects [40]. This version of the classification analysis of variance is robust when statistical assumptions, such as minimal sample size and statistical power, are not satisfied [41]. For this purpose, we considered the main metric of significance, which is the Bayes factor, considering Jeffreys’s [42] cut-offs (≤3 = Anecdotal evidence, between 3 and 10 = Moderate evidence, >10 = Strong evidence). Thus, we considered only differences classified with a strong level of evidence to reject the null hypothesis in a 95% confidence interval [41,42]. The Bayesian ANOVA was performed in R, the statistical computing programming language (version 4.4.2.) [43].

## 3. Results

Table 2 shows the means, standard deviations (mean ± Sd), and percentage of variations for the control (N = 11) and experimental group (N = 13) across two measurement times (M1 and M2) anthropometrics and body composition, physical fitness, and sleep quality. Additionally, it presents the comparisons between moments for the control and experimental groups. In the control group, significant differences were only noted for total sleep (t = 2.869; *p* = 0.017; d = −0.865), indicating a statistically significant decrease in quality. In the experimental group, several variables showed significant differences. The Arm Curl exhibited a significant improvement (t = −4.696; *p* = 0.001; d = −0.373) in the number of arm curls performed. The 5TSTS showed a significant decrease in the time to perform five sit-to-stand movements (t = 5.392; *p* < 0.001; d = −0.058). CS30 had a significant (t = −8.469; *p* < 0.001; d = −0.161) decrease in the number of chair stands performed in 30 s. TUG showed a significant increase in the time to complete the test (t = 4.212; *p* = 0.001; d = 0.243). The Seat and Reach test revealed a decrease in flexibility (t = −4.127; *p* = 0.001; d = −0.265), and the Back Stretch test showed the same tendency (t = −3.722; *p* = 0.003; d = −0.519). The 2MST variable significantly increased the number of steps performed in two minutes (t = −9.617; *p* < 0.001; d = −0.040). The Total Fat (%) significantly decreased (t = 2.225; *p* = 0.046; d = 0.228), and lastly, the Sleep quality score significantly improved (t = 2.856; *p* = 0.014; d = −0.865).

Table 3 below presents the group comparison results after 32 weeks of multicomponent training intervention. The mixed effects Bayesian ANOVA revealed that there were only significant post-prior probabilities (*p* < 0.05) regarding increases in upper limb strength for the experimental group, with a significant isolated effect of time (Bayes Factor = 85.02357 ± 1.12%) and a significant group × time interaction (Bayes Factor = 80.38867 ± 1.76%). There was also a significant increase (*p* < 0.05) in the lower limb flexibility in favour of the experimental group, with only a group × time interaction (Bayes Factor = 7.937376 ± 2.16%). Finally, there was a significant increase in the absolute sleep scores regarding the control group, with a significant group × time interaction (Bayes Factor = 10.34395 ± 2.07%) highlighting the increased risk of sleep disorders in this group.

**Figure 3:**
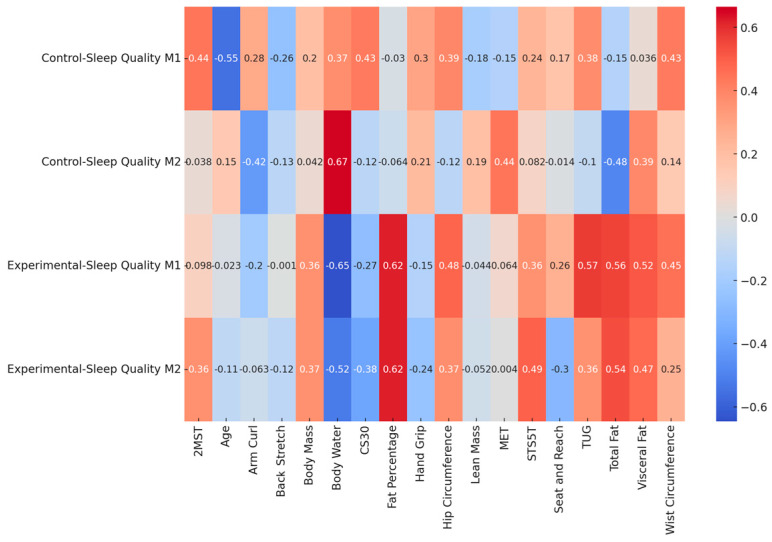
Correlation heat map. Warm colors signify statistically significant associations, and cold and neutral colors represent statistical insignificance.

Intending to understand the link between physical fitness, body composition, and sleep quality, the Pearson correlation test presents significant associations with total sleep (Table 4). The control group revealed significant associations between total sleep quality and body water (Moment 2: r = 0.665; *p* = 0.026). The experimental group revealed significant associations between sleep quality TUG (Moment 1: r = 0.575; *p* = 0.040), total fat (Moment 1: r = 0.526; *p* = 0.046), Fat percentage (Moment 1: r = 0.619; *p* = 0.024 | Moment 2: r = 0.620; *p* = 0.024), and body water (Moment 1: r = 0.646; *p* = 0.017). Figure 3 presents the correlation heatmap.

## 4. Discussion

This study aimed to assess the effects of 8 months of multicomponent training programs on critical variables such as physical fitness, body composition, and sleep quality. It was hypothesized that a multicomponent training program of 8 months significantly improves physical fitness, body composition, and quality of life. The results revealed that after the multicomponent training program, the experimental group improved the arm curl, 5TSTS, CS30, 2MST, total fat (%), and sleep quality; conversely, the TUG, Seat and Reach, and the Back Stretch tests revealed worse results. The control group revealed a worse sleep quality after the follow-up.

Regarding the physical fitness variables, the improvements align with the literature, where multicomponent training programs improve physical fitness and functionality [22,24,30,44,45]. In the current research, the experimental group improved the variables related to aerobic and resistance exercise (arm curl, 5TSTS, CS30, 2MST). The lack of specificity of multicomponent training may explain these results. A previous study [33] revealed that, independent of the training type (multicomponent, resistance, or power), it was possible to note improvements in upper and lower limb strength, upper and lower limb flexibility and aerobic resistance but not in the TUG test after eight months of intervention. Also, after 8 months, a multicomponent training program improved the maximal voluntary contraction of upper and lower limbs in older women [46]. Another study [47] revealed that after 6 months of intervention, the Chair stand test, Arm curl, Chair sit and reach, Back scratch, TUG, 2MST, Hand grip strength, and BMI significantly improved. Regarding the body composition, the EG of the present study reduced the total fat percentage. This aligns with previous studies that applied a multicomponent training program in the older population, where fat mass was reduced after 8 [22] and 6 months [47]. However, there are quite controversial results at 6 months, where in some studies, total fat mass did not reduce after the intervention [46,48]. Furthermore, the multicomponent training programs seem to be adequate to improve aged people’s (>60 years old) physical fitness and frailty [49].

As for the sleep quality, the EG significantly improved the sleep quality. A systematic review with meta-analysis [12] revealed that exercise programs improved sleep quality. The same was noted when applying for multicomponent training programs. In the study by Vaz Fragoso et al. [46], in-home- and center-based participants from 24–30 months with moderate intensity and 5 times per week found sleep quality improvements in the participants. The study from Laredo-Aguilera et al. [50], with 10 weeks of duration and a self-determined intensity to perform 8–12 repetitions, 3 times per week, revealed significant improvements in sleep quality. Finally, Bademli et al. [51], in nursing home residents, applied a 20-week program with moderate intensity and 3–4 times per week and showed improved sleep quality scores. The studies are in line with the current study, where the multicomponent training program with 8-months duration and moderate intensity also improved sleep quality.

The present study evaluated associations between sleep quality, physical fitness, and body composition variables to understand the variables that may explain the total sleep quality for the CG and EG after and before the multicomponent training program. The CG revealed significant associations between total sleep quality and body water (Moment 2: r = 0.665; *p* = 0.026). The association between total sleep quality and body water was noted in the second evaluation moment and revealed that the higher the percentage of body water, the lower the sleep quality. During the night, many elderlies experience the need for frequent urination due to the difficulty of controlling it with aging [52,53,54]. With this need to frequently urinate, the circadian rhythm will negatively impact sleep continuity and quality [54,55]. The EG presented significant associations between sleep quality and TUG (baseline), total fat (baseline), and body water (baseline). The fat percentage presented significant associations with sleep quality at baseline and post-intervention). The results showed that before the multicomponent exercise program, the higher the TUG, the poorer the sleep quality. As for body composition, higher total fat and body water scores also indicate poor sleep quality. Factors such as waist circumference, visceral fat, and total fat can affect sleep quality through hormonal regulation, inflammation, and metabolic health [56,57], possibly affecting the circadian rhythm [54,55] and sleep quality.

Altogether, this study presents promising results regarding the effectiveness of exercise-based interventions to improve physical fitness and sleep quality. However, there are some important limitations to be addressed: (i) the sample of this study is too small and makes it impossible to perform sex comparisons or generalize the results; (ii) the intervention program lasted for 8 months, and daily life activities were not controlled; (iii) co-variates such as mental health or wellbeing behaviors were not assessed; (iv) only two time measures (before and after) were made in this study. Upon that, future research should be conducted: (i) higher sample sizes will allow generalizing of the results; (ii) monitoring daily life physical activity; (iii) mental health and well-being lifestyle should be assessed; (iv) different durations and types of exercise-based programs should be assessed.

## 5. Conclusions

The findings of this study demonstrate that a targeted physical fitness intervention can significantly improve physical performance and sleep quality in aging adults. The experimental group showed marked improvements in muscle endurance, as evidenced by increased arm curl repetitions and improved performance in the 5TSTS and 2MST tests. Additionally, the intervention led to a significant reduction in total fat percentage and enhanced sleep quality. In contrast, the control group exhibited a decline in sleep quality over the study period, highlighting the potential protective effects of physical exercise on sleep in older adults. These results underscore the importance of incorporating regular, targeted exercise programs into the routines of aging individuals to enhance overall health, physical fitness, and sleep quality.

## Figures and Tables

**Table 1:** Components and exercises were used day-by-day over the week for the training sessions during the training protocol.

Components	Day #1 of the Week	Day #2 of the Week	Day #3 of the Week
**Warm-up** **(5 min)**	Jogging and cardio-based warm-up Dynamic stretching targeting shoulders, hips, and ankles.	Jogging and cardio-based warm-up Dynamic stretching targeting shoulders, hips, and ankles.	Jogging and cardio-based warm-up Dynamic stretching targeting shoulders, hips, and ankles.
**Resistance training** **(1–3 sets; 15–20 min)**	**1–3 sets, 40–60 s rest between sets** 6 repetitions (reps) kettlebell clean and press + 6 reps single-arm kettlebell thrusters 12 reps single-arm kettlebell rows 12 reps kettlebell sumo deadlift high pulls 12 reps dumbbell lateral raises 12 reps single-arm kettlebell triceps extensions	**1–3 sets, 40–60 s rest between sets** 12 reps alternating single-arm kettlebell rows (touching the ground) 12 reps Romanian deadlifts 12 reps Dumbbell chest flys 12 reps single-arm kettlebell curls 12 reps kettlebell biceps curls	**1–3 sets, 40–60 s rest between sets** 12 reps single-arm KB swings per side 12 reps KB goblet squats: focus on a slow descent, explosive ascent 12 reps bodyweight lunges: with a 2-s pause at the bottom 12 reps KB Romanian deadlifts 6 reps KB overhead extensions 6 reps triceps kickbacks per side
**Balance training** **(5–8 min)**	**(2 sets, IR: 30 s between sets)** Single-leg deadlifts: 3 per leg Dynamic lateral lunges: 3 per side (hold each lunge position for 3 s) Alternating high knees: 3 per side (hold each knee up for 3 s) Reverse lunges with glute squeeze: 6 per leg Toe touches with step forward: 6 per side (step forward to touch toes)	**(2 sets, IR: 30 s between sets)** 5 m side shuffle + 2 alternating side lunges + 5 m Jog 10 m heel-to-toe walk 6 high knees with 2 quick taps	**(2 sets, IR: 30 s between sets)** Complex [4 high knees with hold + 4 controlled leg swings] 4 toe taps with ankle mobility + 4 static high knees Single-leg complex [2 controlled leg swings + 2 quick high knees + 2 single-leg balance holds (without touching the ground)]
**Aerobic fitness** **15–20 min)**	**3 Sets, 60 s ON, 30 s OFF, IR: 60 s between sets** Marching in place Light jogging Light jogging Step touches Low-impact jumping jacks	**3 Sets, 60 s ON, 30 s OFF, IR: 60 s between sets** **Arm circles** Punches (shadow boxing) Shoulder taps Front and lateral raises High knees with arm swing	**3 Sets, 60 s ON, 30 s OFF, IR: 60 s between sets** High knees Arm circles Step touches Lateral lunges Front and lateral raises
**Cool down** **(5 min)**	**5 min** Upper and lower body static Stretching: stretches like hamstring stretches, quadriceps stretches, shoulder stretches, and tricep stretches. Dynamic trunk stretching and breathing exercises: torso twists, side bends, and deep diaphragmatic breathing.	**5 min** Upper and lower body static stretching: stretches like hamstring stretches, quadriceps stretches, shoulder stretches, and tricep stretches. Dynamic trunk stretching and breathing exercises: torso twists, side bends, and deep diaphragmatic breathing.	**5 min** Upper and lower body static stretching: stretches like hamstring stretches, quadriceps stretches, shoulder stretches, and tricep stretches. Dynamic trunk stretching and breathing exercises: torso twists, side bends, and deep diaphragmatic breathing.

**Table 2:** Mean, standard deviations, percentage of variations, and comparisons for anthropometrics, body composition, physical fitness, and sleep quality measures for the control and experimental groups by moments of evaluations.

	Control Group (N = 11)						Experimental Group (N = 13)					
	M1 Mean ± Sd	M2 Mean ± Sd	Δ%	t	*p*	d	M1 Mean ± Sd	M2 Mean ± Sd	Δ%	t	*p*	d
Body Mass (Kg)	65.64 ± 6.77	66.00 ± 5.44	0%	−0.251	0.807	−0.068	63.62 ± 12.99	64.75 ± 11.88	3%	−0.794	0.443	−0.068
Hang Grip (Kgf)	26.91 ± 7.83	26.72 ± 5.83	0%	0.110	0.915	−0.007	20.08 ± 8.11	22.50 ± 5.64	12%	−1.351	0.202	−0.007
Arm Curl (Reps)	22.27 ± 5.42	24.09 ± 3.24	8%	−1.237	0.244	−0.373	18.92 ± 3.01	24.31 ± 2.59	26%	−4.696	0.001 *	−0.373
Waist circumference (cm)	89.73 ± 7.18	85.73 ± 8.71	−4%	1.298	0.223	0.391	85.35 ± 11.82	84.79 ± 11.40	0%	0.638	0.535	0.391
Hip circinferemce (cm)	101.82 ± 6.74	101.36 ± 3.34	0%	0.255	0.804	0.084	98.46 ± 9.98	98.38 ± 10.44	0%	0.166	0.871	0.084
5TSTS (sg)	6.85 ± 1.16	6.93 ± 1.43	1%	−0.155	0.880	−0.058	7.54 ± 1.11	6.10 ± 1.24	−18%	5.392	<0.001 *	−0.058
CS30 (reps)	22.73 ± 2.94	23.27 ± 4.47	2%	−0.534	0.605	−0.161	23.85 ± 4.51	20.31 ± 3.99	19%	−8.469	<0.001 *	−0.161
TUG (sg)	5.91 ± 0.701	5.41 ± 1.33	−7%	1.025	0.330	0.243	4.79 ± 0.68	5.56 ± 1.02	−14%	4.212	0.001 *	0.243
Seat and Reach (cm)	3.27 ± 5.35	6.73 ± 9.63	106%	−0.879	0.400	−0.265	−6.52 ± 11.18	2.15 ± 6.73	−118%	−4.127	0.001 *	−0.265
Back Stretch (cm)	−7.46 ± 10.69	−4.45 ± 9.32	−40%	−1.722	0.116	−0.519	−6.89 ± 8.52	−3.46 ± 7.63	−52%	−3.722	0.003 *	−0.519
2MST (reps)	189.18 ± 38.19	192.64 ± 62.22	2%	−0.134	0.896	−0.040	174.69 ± 26.85	222.08 ± 37.17	29%	−9.617	<0.001 *	−0.040
Total Fat (kg)	20.55 ± 5.20	19.73 ± 4.43	−4%	0.726	0.484	0.213	20.24 ± 7.15	19.59 ± 7.71	−3%	1.888	0.083	0.213
Total Fat (%)	30.91 ± 5.69	29.81 ± 4.77	−3%	0.815	0.434	0.228	29.42 ± 8.82	26.61 ± 9.73	−10%	2.225	0.046 *	0.228
Lean Mass (kg)	43.09 ± 4.59	43.00 ± 3.77	−1%	0.115	0.911	0.094	41.97 ± 6.13	42.40 ± 5.65	1%	−0.758	0.463	0.094
Body Water (%)	48.73 ± 4.03	48.55 ± 3.98	0%	0.176	0.864	−0.003	49.38 ± 5.55	49.95 ± 5.69	1%	−1.171	0.264	−0.003
Visceral Fat (a.u.)	7.91 ± 2.59	8.18 ± 2.52	3%	−1.399	0.192	−0.422	7.08 ± 2.63	6.92 ± 2.33	−1%	0.519	0.613	−0.422
MET (Kcal)	1341.64 ± 125.61	1356.91 ± 93.91	−19%	1.384	0.196	0.417	1304.08 ± 183.17	1310.54 ± 181.06	1%	−0.567	0.581	0.417
Sleep Quality (a.u.)	4.64 ± 2.34	6.82 ± 1.17	47%	−2.869	0.017 *	−0.865	5.46 ± 1.45	4.62 ± 1.45	−14%	2.856	0.014 *	−0.865

Legend: Kg—kilograms; Kgf—Kilograms of force; reps—repetitions; 5TSTS—Five Times Sit-to-Stand Test; CS30—30-Second Chair Stand Test; TUG—Timed Up and Go Test; 2MST—Two-Minute Step Test; MET—Metabolic Rate; * *p* < 0.05.

**Table 3:** Results of 32 weeks of multicomponent training in the functional fitness and body composition regarding the interaction with group and time.

Variable	Moment	Exercise (N = 13)	Control (N = 11)	ANOVA	Bayes Factor	Sig. Prob.
Body mass (kg)	Pre	63.6 ± 12.9	65.6 ± 6.77	Time	0.31 ± 4.84%	Anecdotal
	Post	64.6 ± 11.8	66 ± 5.44	Group	0.38 ± 1.1%	Anecdotal
				Interaction	0.04 ± 1.65%	Anecdotal
HG (kgf)	Pre	20.2 ± 8.18	26.9 ± 7.83	Time	0.37 ± 0.98%	Anecdotal
	Post	22.6 ± 5.68	26.7 ± 5.83	Group	2.41 ± 0.95%	Anecdotal
				Interaction	0.44 ± 2.48%	Anecdotal
ULS (rep)	Pre	18.9 ± 3.01	22.3 ± 5.42	Time	85.02 ± 1.12%	Strong
	Post	24.3 ± 2.59	24.1± 3.24	Group	0.56 ± 0.64%	Anecdotal
				Interaction	80.39 ± 1.76%	Strong
Waist (cm)	Pre	85.5 11.8	89.9 ± 7.20	Time	0.44 ± 0.86%	Anecdotal
	Post	84.8 11.4	85.7 ± 8.72	Group	0.39 ± 3.49%	Anecdotal
				Interaction	0.08 ± 2.13%	Anecdotal
Hip (cm)	Pre	98.5 ± 9.94	102.0 ± 6.74	Time	0.29 ± 1.32%	Anecdotal
	Post	98.4 ± 10.5	101.0 ± 3.36	Group	0.57 ± 1.7%	Anecdotal
				Interaction	0.06 ± 2.09%	Anecdotal
LLP (sec)	Pre	7.46 ± 1.33	6.73 ± 1.35	Time	0.85 ± 0.88%	Anecdotal
	Post	6.08 ± 1.26	7.09 ± 1.3	Group	0.37 ± 0.81%	Anecdotal
				Interaction	2.00 ± 3.31%	Anecdotal
LLS (rep)	Pre	20.3 ± 3.99	22.7 ± 2.94	Time	1.92 ± 1.74%	Anecdotal
	Post	23.8 ± 4.51	23.3 ± 4.47	Group	0.43 ± 0.8%	Anecdotal
				Interaction	0.78 ± 2.95%	Anecdotal
DB (sec)	Pre	5.56 ± 1.02	5.80 ± 0.824	Time	2.68 ± 0.95%	Anecdotal
	Post	4.78 0.681	5.38 ± 1.34	Group	0.61 ± 2.98%	Anecdotal
				Interaction	0.79 ± 3.6%	Anecdotal
LLF (cm)	Pre	−6.54 ± 11.2	3.27 ± 5.35	Time	3.79 ± 0.64%	Moderate
	Post	2.15 ± 6.73	6.73 ± 9.63	Group	2.62 ± 0.63%	Anecdotal
				Interaction	7.94 ± 2.16%	Strong
ULF (cm)	Pre	−6.85 ± 8.57	−7.46 ± 10.7	Time	0.82 ± 0.72%	Anecdotal
	Post	−3.46 ± 7.63	−4.46 ± 9.32	Group	0.41 ± 0.56%	Anecdotal
				Interaction	0.80 ± 83.77%	Anecdotal
AF (rep)	Pre	175.0 ± 26.9	189.0 ± 38.2	Time	2.33 ± 0.99%	Anecdotal
	Post	222.0 ± 37.2	193.0 ± 62.2	Group	0.36 ± 0.75%	Anecdotal
				Interaction	1.24 ± 1.78%	Anecdotal
Tot. BF (kg)	Pre	20.3 ± 7.05	20.5 ± 5.20	Time	0.31 ± 2.31%	Anecdotal
	Post	19.7 ± 7.74	19.7 ± 4.43	Group	0.36 ± 0.94%	Anecdotal
				Interaction	0.04 ± 3.85%	Anecdotal
BF percentage (%)	Pre	29.4 ± 8.80	30.9 ± 5.63	Time	0.46 ± 1.09%	Anecdotal
	Post	26.5 ± 9.81	29.9 ± 4.66	Group	0.52 ± 1.41%	Anecdotal
				Interaction	0.10 ± 5.03%	Anecdotal
Tot. LM (kg)	Pre	42 ± 6.03	43.3 ± 4.65	Time	0.30 ± 1.29%	Anecdotal
	Post	42.4 ± 5.74	43 ± 3.77	Group	0.45 ± 7.9%	Anecdotal
				Interaction	0.05 ± 2.41%	Anecdotal
Wat. Percentage (%)	Pre	50 ± 5.13	48.7 ± 4.03	Time	0.29 ± 1.49%	Anecdotal
	Post	50.1 ± 5.75	48.7 ± 4.12	Group	0.50 ± 2.21%	Anecdotal
				Interaction	0.05 ± 2.3%	Anecdotal
Visc. Fat (Index)	Pre	7.08 ± 2.63	7.91 ± 2.59	Time	0.30 ± 1.65%	Anecdotal
	Post	6.92 ± 2.33	8.18 ± 2.52	Group	0.64 ± 0.98%	Anecdotal
				Interaction	0.07 ± 2.43%	Anecdotal
Basal Met.	Pre	1304 ± 183	1669 ± 762	Time	0.58 ± 1.55%	Anecdotal
	Post	1311 ± 181	1357 ± 937	Group	0.83 ± 0.78%	Anecdotal
				Interaction	0.47 ± 1.64%	Anecdotal
Sleep score	Pre	5.46 ± 1.45	4.64 ± 2.34	Time	0.51 ± 3.41%	Anecdotal
	Post	4.62 ± 1.26	6.82 ± 1.17	Group	0.56 ± 1.11%	Anecdotal
				Interaction	10.35 ± 2.07%	Strong

**Table 4:** Associations of sleep quality with anthropometrics, body composition, physical fitness variables, and sleep quality for both control and experimental groups, and by moments.

Group	Variable		Age	Body Mass	Hand Grip	Arm Curl	Wist Circumference	Hip Circumference	STS5T	CS30	TUG	Seat and Reach	Bach Strech	2MST	Total Fat	Fat Percentage	Lean Mass	Body Water	Visceral Fatt	MET
Control	Sleep Quality M1	r	−0.55	0.196	0.3	0.282	0.427	0.392	0.237	0.426	0.378	0.175	−0.255	0.441	−0.153	−0.03	−0.183	0.372	0.036	−0.155
		*p*	0.079	0.564	0.37	0.402	0.19	0.233	0.483	0.191	0.252	0.606	0.448	0.174	0.654	0.929	0.59	0.26	0.917	0.649
	Sleep Quality M2	r	0.150	0.042	0.214	−0.418	0.145	−0.124	0.082	−0.124	−0.101	−0.014	−0.128	0.038	−0.485	−0.064	0.193	0.665 *	0.386	0.442
		*p*	0.659	0.901	0.528	0.201	0.670	0.716	0.810	0.717	0.768	0.968	0.708	0.913	0.130	0.833	0.623	0.026	0.241	0.174
Experimental	Sleep Quality M1	r	−0.023	0.364	−0.154	−0.201	0.454	0.485	0.360	−0.272	0.575 *	0.256	−0.001	0.098	0.562 *	0.619 *	−0.044	−0.646 *	0.515	0.064
		*p*	0.941	0.221	0.616	0.510	0.119	0.093	0.227	0.369	0.040	0.399	0.997	0.750	0.046	0.024	0.886	0.017	0.072	0.837
	Sleep Quality M2	r	−0.113	0.367	−0.243	−0.063	0.253	0.368	0.492	−0.378	0.359	−0.297	−0.115	0.363	0.541	0.620 *	−0.052	−0.523	0.472	0.004
		*p*	0.714	0.218	0.423	0.839	0.404	0.217	0.088	0.203	0.229	0.325	0.708	0.222	0.056	0.024	0.867	0.067	0.103	0.991

Legend: 5TSTS—Five Times Sit-to-Stand Test; CS30—30-Second Chair Stand Test; TUG—Timed Up and Go Test; 2MST—Two-Minute Step Test; MET—Metabolic Rate; * *p* < 0.05.

## Data Availability

Contact corresponding author.
